# Isolation of human genomic DNA for genetic analysis from premature neonates: a comparison between newborn dried blood spots, whole blood and umbilical cord tissue

**DOI:** 10.1186/1471-2156-14-105

**Published:** 2013-10-29

**Authors:** Shavanthi Rajatileka, Karen Luyt, Manal El-Bokle, Maggie Williams, Helena Kemp, Elek Molnár, Anikó Váradi

**Affiliations:** 1 Centre for Research in Biosciences, Department of Biological, Biomedical and Analytical Sciences, Faculty of Health and Applied Sciences, University of the West of England, Bristol BS16 1QY, UK; 2 Neonatal Neuroscience, School of Clinical Sciences, University of Bristol, St Michael’s Hospital, Southwell Street, Bristol BS2 8EG, UK; 3 Southmead Hospital, Bristol BS10 5NB, UK; 4 Bristol Genetics Laboratory, Pathology Sciences, Blood Sciences and Bristol Genetics, Southmead Hospital, Bristol BS10 5NB, UK; 5 Department of Chemical Pathology, Southmead Hospital, Bristol BS10 5NB, UK; 6 Centre for Synaptic Plasticity, School of Physiology and Pharmacology, University of Bristol, Medical Sciences Building, University Walk, Bristol BS8 1TD, UK

**Keywords:** Premature newborns, Single nucleotide polymorphism, Newborn dried blood spots, Umbilical cord, Genomic DNA, Pyrosequencing, Sample storage

## Abstract

**Background:**

Genotyping requires biological sample collection that must be reliable, convenient and acceptable for patients and clinicians. Finding the most optimal procedure of sample collection for premature neonates who have a very limited blood volume is a particular challenge. The aim of the current study was to evaluate the use of umbilical cord (UC) tissue and newborn dried blood spot (DBS)-extracted genomic DNA (gDNA) as an alternative to venous blood-derived gDNA from premature neonates for molecular genetic analysis.

All samples were obtained from premature newborn infants between 24-32 weeks of gestation. Paired blood and UC samples were collected from 31 study participants. gDNA was extracted from ethylenediaminetetraacetic acid (EDTA) anticoagulant-treated blood samples (~500 μl) and newborn DBSs (n = 723) using QIAamp DNA Micro kit (Qiagen Ltd., Crawley, UK); and from UC using Qiagen DNAeasy Blood and Tissue kit (Qiagen Ltd., Crawley, UK). gDNA was quantified and purity confirmed by measuring the A_260_:A_280_ ratio. PCR amplification and pyrosequencing was carried out to determine suitability of the gDNA for molecular genetic analysis. Minor allele frequency of two unrelated single nucleotide polymorphisms (SNPs) was calculated using the entire cohort.

**Results:**

Both whole blood samples and UC tissue provided good quality and yield of gDNA, which was considerably less from newborn DBS. The gDNA purity was also reduced after 3 years of storage of the newborn DBS. PCR amplification of three unrelated genes resulted in clear products in all whole blood and UC samples and 86%-100% of newborn DBS. Genotyping using pyrosequencing showed 100% concordance in the paired UC and whole blood samples. Minor allele frequencies of the two SNPs indicated that no maternal gDNA contamination occurred in the genotyping of the UC samples.

**Conclusions:**

gDNAs from all three sources are suitable for standard PCR and pyrosequencing assays. Given that UC provide good quality and quantity gDNA with 100% concordance in the genetic analysis with whole blood, it can replace blood sampling from premature infants. This is likely to reduce the stress and potential side effects associated with invasive sample collection and thus, greatly facilitate participant recruitment for genetic studies.

## Background

The reliability and performance of the molecular assays such as polymerase chain reaction (PCR) and pyrosequencing are strongly influenced by the quality and quantity of the starting template. The availability of high quality gDNA from a large number of well characterised patients and healthy controls is a prerequisite for the success of genetic variation studies. Conventionally, gDNA for use in clinical epidemiological studies is obtained from peripheral blood samples because it provides high quality and a good yield of gDNA [1-4]. However, obtaining peripheral blood is invasive and unsuitable for certain cohorts such as very low birthweight preterm infants because they have a small circulating blood volume (~85 ml/kg); [5-7] and it is not considered to be ethical to sample more than 1 ml for research purposes. Recalling these infants at a later stage when suitable amount of whole blood can be collected is problematic because the neonatal mortality rate in the very low birthweight cohort is significant, particularly in the high risk preterm group [8].

An alternate source of gDNA, which is now used frequently in molecular genetic studies, is newborn dried blood spots (DBS) [9-13]. The blood is usually collected by a heel-prick and applied on special filter paper, a convenient medium for transport and storage [14]. These newborn DBS are used for neonatal metabolic screening and then stored in repositories for follow-up testing and public health research [15-18]. Using newborn DBS would be an ideal replacement for the use of fresh human tissue for gDNA extraction, as it is routinely carried out at birth eliminating the need for additional needle pricks for sample collection and for specialist storage conditions. The drawback of using newborn DBS for genetic analysis is the miniscule amount of blood available. The amount of gDNA that can be extracted from a 3.2 mm punch of a newborn DBS is about 60 ng [19]. In reality, only about one to maximum three 3.2 mm punches are available for academic research purposes, which is not sufficient for large scale single nucleotide polymorphisms (SNP) detection studies.

This problem can be overcome by using umbilical cord blood, aspirated from the placenta after birth, for gDNA preparation. The practice of delayed cord clamping is advantageous to the preterm infant [20-22], facilitating an autotransfusion from the placenta. However, this means that the volume of infant blood remaining in the cut umbilical cord and placenta is significantly reduced. Cord blood is frequently required for clinical indications, such as blood group haemoglobin and serum bilirubin analysis, taking priority over research samples. When cord blood is aspirated, there is also a potential risk of contamination by maternal blood [23]. However, umbilical cord tissue which would usually be discarded as clinical waste following birth [24] can be collected easily and potentially used for gDNA extraction [25].

In this study we compared three different sources for gDNA extraction from very premature babies where a large volume of whole blood or umbilical cord blood is not available. The suitability of newborn DBS and umbilical cord tissue for PCR and pyrosequencing was investigated and the concordance of paired umbilical cord tissue gDNA and whole blood from the same individual was assessed. Our study showed that umbilical cord tissue can effectively be used for genetic analysis of premature babies.

## Methods

### Sample collection and processing

Blood, newborn DBS and umbilical cord tissue were collected from a subset of patients participating in an association study to investigate the genetic background of premature infants to white matter brain injury. The study received ethical approval in April 2008 from the National Research Ethics Service, UK (REC reference number 10/H0106/10). For the use of whole blood and umbilical cord written informed consent was obtained from the parents of eligible infants participating in the study. Similarly, informed written consent was obtained from healthy adult volunteers for the use of whole blood samples. The archived newborn blood spot samples used for the study were fully anonymised according to the Human Tissue Act and MRC Guidance and used for research without individual informed consent as permitted by the UK newborn screening programme Code of Practice for the retention and Storage of Residual Spots (April 2005, ISBN 0955013801).

### Blood samples

Whole blood samples (~500 μl) were obtained from preterm infants between 24-32 week gestation during the first week of life when stable on intensive care. Samples were collected in K_2_-EDTA tubes, mixed by inversion 8-10 times after being drawn and stored at 4°C for up to a month prior to gDNA isolation.

### Dried blood spots (DBS)

Newborn DBS were collected from heel prick blood sampling on blood spot screening cards prepared routinely within 5-8 days of birth as part of the UK Newborn Screening Programme [http://newbornbloodspot.screening.nhs.uk]. Samples, collected from infants 24-32 weeks gestation within the past 3-22 years, were used in the study. The newborn DBS samples from the participants that were stored in the biobank were 3-5 years old (n = 25); 6-10 years old (n = 25); 11-15 years old (n = 20); and 16-22 years old (n = 30). Newborn DBS obtained within the last three years were not available for analysis because these samples might need to be recalled by the pathology laboratories for further tests for up to 3 years after birth. All blood spot screening cards were stored in boxes at room temperature. To compare yields and quality from more recently prepared dried blood spots, 25 μl of whole blood from volunteer adults was spotted onto a blood spot screening card, air dried and stored at room temperature for a period of one month (n = 20).

### Umbilical cord tissue

**Figure 1:**
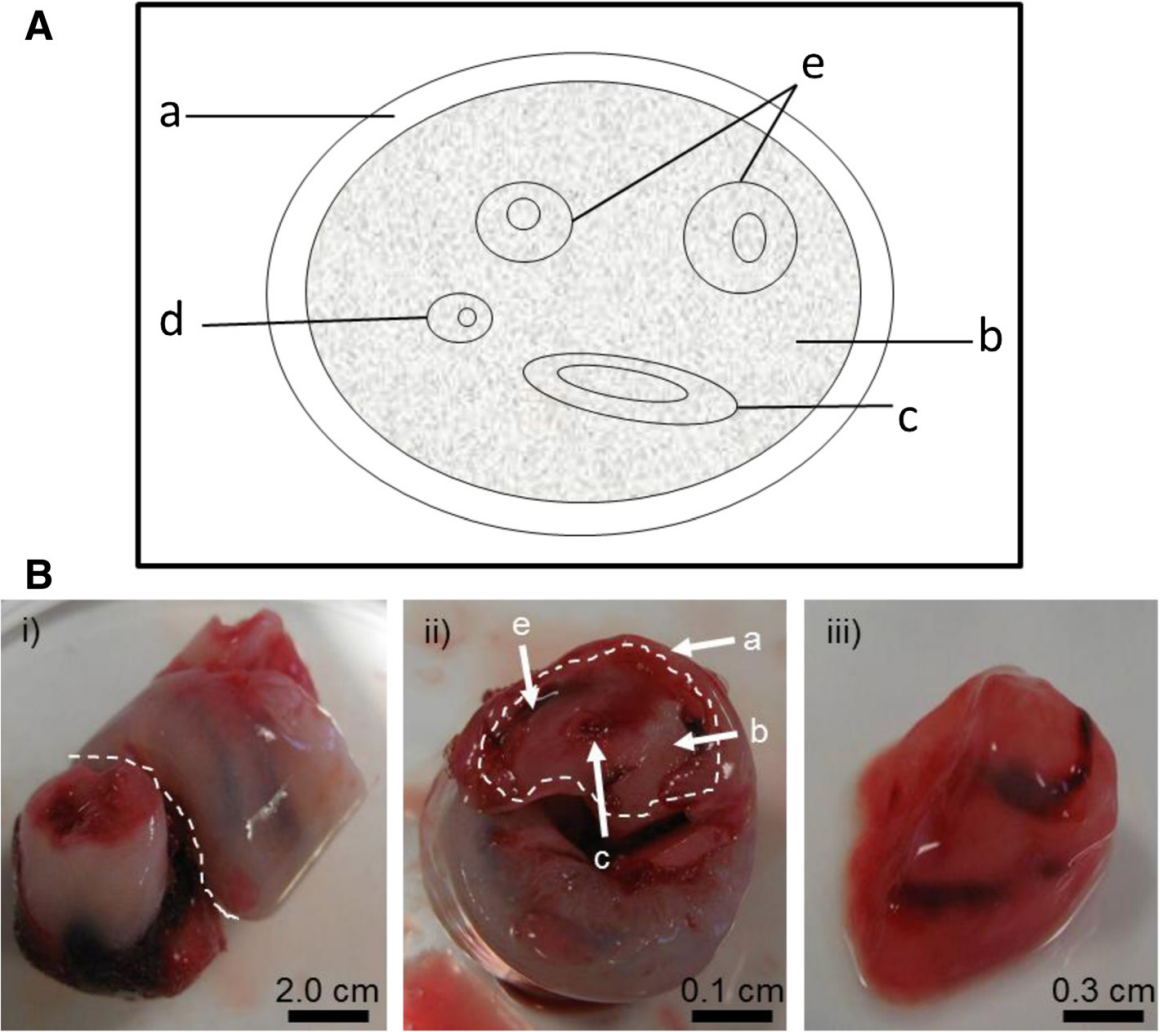
**Isolation of Umbilical cord tissue for DNA extraction. (A)** A schematic structure of the umbilical cord (cross sectional view). a maternal sheath, b Wharton’s jelly; c umbilical vein; d allantoic duct; e umbilical arteries. **(B)** Umbilical cord preparation for gDNA extraction. i) Cord tissue was cut across as indicated with the white line. ii) Cross-section of the umbilical cord. The outer maternal sheath was removed as indicated with the white line. iii) The internal Wharton’s jelly with umbilical vein and arteries was used for gDNA extraction.

A 5-10 cm long segment collected from the mid portion of each cord was obtained immediately following delivery, washed in sterile water and stored in sterile 30 ml specimen containers at−20°C until required for DNA isolation (Figure 1).

### DNA isolation

DNA from 100 μl of whole blood and all newborn DBS was isolated using the QIAamp DNA Micro Kit (QIAGEN Ltd., Crawley, UK) following manufacturer’s guidelines. For newborn DBS, one to three 3.2 mm disks were punched from each card for DNA extraction. Umbilical cord DNA was extracted from the inner layer of the cord tissue including the Wharton’s jelly and blood vessels (0.5 g wet tissue) using the DNeasy Blood & Tissue Kit (QIAGEN Ltd., Crawley, UK) according to the manufacturer’s instructions. Umbilical cords were thawed for 5 min at room temperature (~20°C), and during the thawing whilst semi frozen, the outer layer (Figure 1A&B) was removed with a sterile scalpel. This was done to prevent cross-contamination of the infant genomic DNA with maternal or other external DNA due to handling following birth. 0.5 g of the inner layer of the cord tissue including the Wharton’s jelly and blood vessels were used for DNA extraction (Figure 1A&B). DNA was stored at −20°C until analysis. The key steps of the DNA isolation protocols are summarised in Table 1.

**Table 1:** Comparison of the key steps of gDNA extraction protocols from the three different starting materials

**DNA isolation step**	**Starting material**		
	**3.2 mm DBS**	**100 μl WB**	**0.5 g UC**
**1. DNA binding column**	Silica-based	Silica-based	Silica-based
2. Use of carrier RNA*	Yes	Yes	No
**3. Duration of lysis step**	1 h	1 h	~12 h
**4. Elution volume**	60 μl	100 μl	1 ml
**5. Solution used for elution**	Nuclease free water	Nuclease free water	Nuclease free water

*Carrier RNA was added into the lysis buffer at manufacturer’s recommended concentrations (DBS: 0.01 μg/μl and WB: 0.005 μg/μl). DBS–dried blood spots; WB–whole blood; UC–umbilical cord.

### gDNA quantification

DNA concentration was measured at 260 nm against nuclease free water using a NanoDrop ND-1000 (Labtech International Ltd, Ringmer East Sussex, UK). The purity of gDNA was determined by measuring the 260-280 nm absorbance ratio (A_260_:A_280_; Table 2). Optimal purity is expected to be in the range of 1.7-2.0.

**Table 2:** gDNA concentration measured at 260 nm

**Sample**	**DBS**					**DBS average**	**WB**	**UC**
**Storage duration**	**1 month**	**3-5 years**	**6-10 years**	**11-15 years**	**15-22 years**	**3-22 years**	**(<1 month)**	**(<1 month)**
	**n = 20**	**n = 25**	**n = 25**	**n = 15**	**n = 30**	**n = 95**	**n = 31**	**n = 31**
ng/μl	9.2 ± 1.5	6.2 ± 8.7	11.4 ± 6.9	7.9 ± 2	7.6 ± 4.2	8.3 ± 10.7	40.3 ± 10.9	117.3 ± 112.9
Total DNA (μg)	0.55	0.37	0.68	0.48	0.46	0.49	4.03	70.4
A_260_:A_280_	1.7	2.0	2.2	2.5	2.4	2.3	1.8	1.9

p < 0.01 WB *vs* UC, p < 0.001 DBS (3-22 years average) *vs* UC total DNA.

### gDNA quality assessment by gel electrophoresis

The integrity of the gDNA samples were assessed by analysing the samples (10-50 ng) for evidence of degradation using agarose gel electrophoresis. Genomic DNA samples were run on an agarose gel (0.75% agarose) containing 0.5 μg/ml ethidium bromide alongside a DNA ladder, lamda-Hind*III* (Thermoscientific, Massachusetts, USA) for 90 min. Samples were visualised under ultraviolet light (Gel Doc 1000, Bio Rad Laboratories Ltd, Hemel Hempstead, UK). The size of the gDNA was determined by comparison with the DNA ladder. Appropriate quality gDNA is expected to migrate predominantly above10 kb on agarose gels.

### Assessment of genomic DNA by PCR amplification

To evaluate the gDNA quality, PCR amplification was performed first on two randomly selected samples from each group of DNA source and from each storage length, using primers for human β-actin (GeneBank accession number X00351) a house-keeping gene with the following primers 5′-TGCCCATCTACGAGGGGTATG-3′ and 5′-GAAATCGTGCGTGACATTAAGGAG-3′. To compare amplification rates for gDNA extracted from different sources (whole blood (n = 31), umbilical cord (n = 31) and newborn DBS (n = 723)), amplification was also carried out flanking two unrelated SNPs: rs1835740 [26] and rs4354668 [27]. All PCR assays were carried out for 35 cycles in a total volume of 25 μl, containing 1× high fidelity reaction buffer - (100 mM Tris–HCl, 500 mM KCl pH 8.3), 1 mM of MgCl_2_, 200 μM of each dNTP, 100 pmol of each oligonucleotide primer, 1 unit of high fidelity Taq Polymerase (FastStart High Fidelity Taq Polymerase, Roche Diagnostics Limited, West Sussex, UK) and 2 μl (~1-30 ng) of gDNA.

### Assessment of the fidelity of gDNA obtained from umbilical cords

To assess the fidelity of the gDNA obtained from umbilical cords, two single nucleotide polymorphisms (SNPs) rs1835740 [26] and rs4354668 [27] were genotyped by pyrosequencing (Qiagen Ltd., Crawley, UK) using paired gDNA isolated from both whole blood and umbilical cords from the same individual (n = 31).

### Pyrosequencing

Single-stranded biotinylated PCR products were prepared for the pyrosequencing reaction using a Vacuum Prep Tool (Qiagen Ltd., Crawley, UK). The biotinylated PCR products were immobilised onto high performance streptavidin sepharose beads (Streptavidin Sepharose™ HP, GE Healthcare, Chalfont St Giles, Buckinghamshire, UK). For a single sample, 3 μl of streptavidin sepharose were added to 40 μl binding buffer (10 mM Tris–HCl, 2 M NaCl, 1 mM EDTA, 0.1% TweenTM 20, pH 7.6; Qiagen Ltd., Crawley, UK) and mixed with 20 μl PCR product and 17 μl deionised water on a mechanical shaker for 5 min at room temperature (~20°C) in a 96-well plate. The beads containing the immobilised templates were isolated by filter probes using vacuum and then washed with 70% ethanol, denaturizing solution (0.2 M NaOH; Qiagen Ltd., Crawley, UK) and then washing buffer (10 mM Tris-acetate pH 7.6; Qiagen Ltd., Crawley, UK) for 5 s each. Beads were released into a PSQTM 96 well plate (Qiagen Ltd., Crawley, UK) containing 38.4 μl annealing buffer (20 mM Tris-acetate, 5 mM magnesium acetate, pH 7.6; Qiagen Ltd., Crawley, UK) and 1.6 μl of the sequencing primer, rs1835740PyroSeq (0.4 μM final concentration). Annealing was achieved by heating the samples to 80°C for 5 min followed by cooling to room temperature (~20°C). Pyrosequencing reactions were performed on the PyroMark™ Q96 ID (Qiagen Ltd., Crawley, UK) according to the manufacturer’s instructions using the PSQTM 96 SNP Reagent Kit (Qiagen Ltd., Crawley, UK; Table 3) and the genotype was determined using PyroMark™ ID program (Qiagen Ltd., Crawley, UK).

**Table 3:** Pyrosequencing primers and conditions used in the study

**Oligonucleotide**	**Sequence 5′-3′**	**Product size (bp)**	**T (°C)**	**Modifications**
rs1835740PyroF	CTCATTCGTTTTCTGCCTGTTG	300	60	None
rs1835740PyroR-BIO	TCTTGCATATTTGAGCAGACTTTG			5′Biotin
rs1835740PyroSeq	CACAACTTGATTCCAATCT	N/A		None
Target sequence	G**C/T**GTATGTAGATT			
Nucleotide dispensation order	AGCTCGTAT			
rs4354668PyroF-BIO	GGGGCTAAACCTTGCAATC	166	60	5′Biotin
rs4354668PyroR	GAGTGGCGGGAGCAGAGA			None
rs4354668PyroSeq	GGGTGTGTGCGCGCC	N/A		None
Target sequence	**T/G**GGGGAGGCGGTGGAGGCC			
Nucleotide dispensation order	CGTGCAGCGTGAGCGTGC			

**Figure 2:**
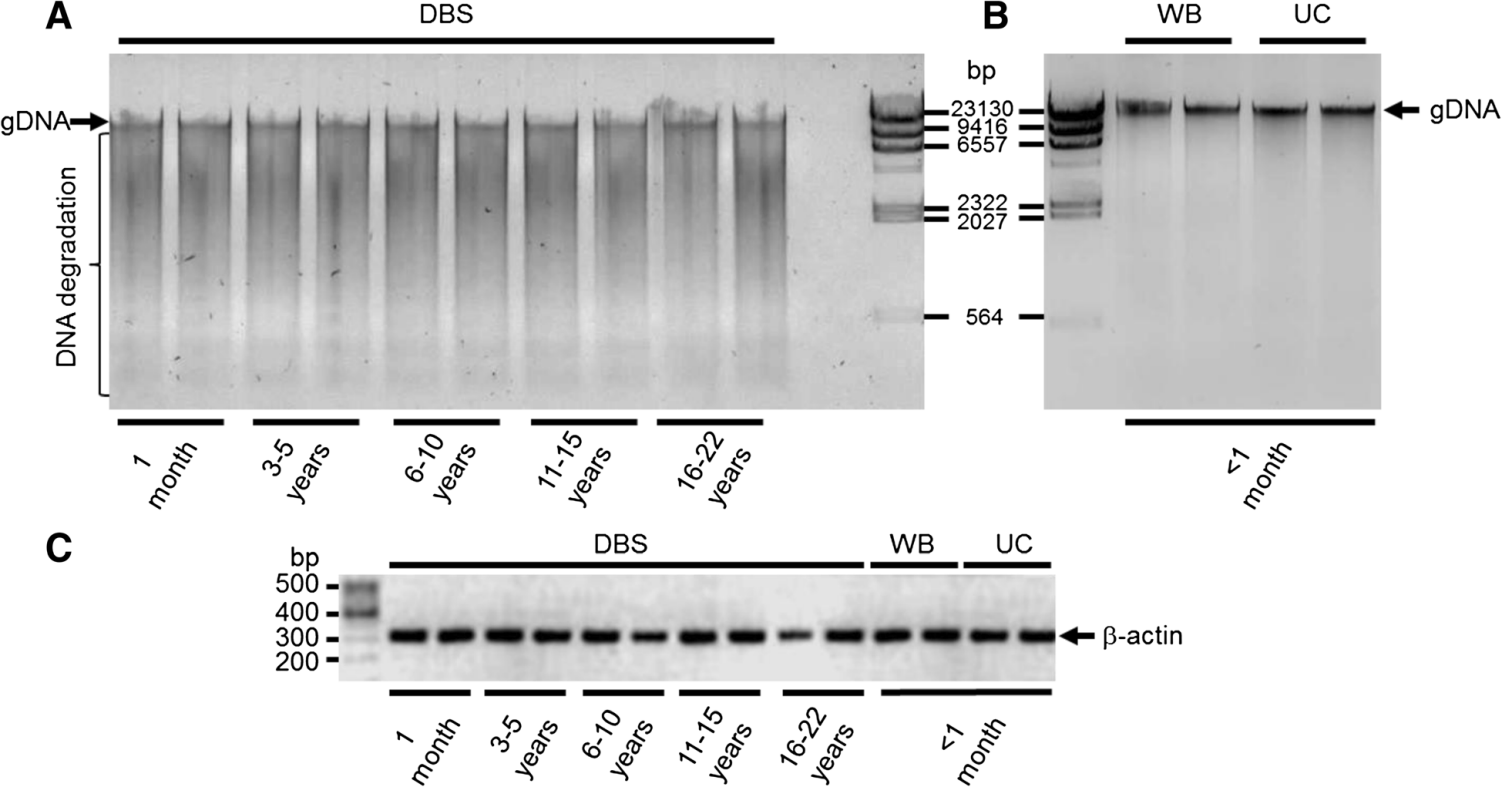
**Agarose gel analysis of gDNA isolated from DBS (A), whole blood (WB) and umbilical cord (UC; B) samples.** Lamda-Hind*III* marker was used as indicated. For DBS 10 ng of gDNA and for WB and UC 50 ng of gDNA were loaded. PCR amplification of the human β-actin gene (325 bp) is shown **(C)**. 100 bp marker (Thermo Fisher Scientific, Hemel Hempstead, UK) is indicated on the left. Equal volumes of PCR reactions were loaded on a 2% TAE agarose gel. Images were inverted using Adobe Photoshop™ to highlight details.

Primer pair rs1835740PyroF/rs1835740PyroR-BIO and rs4354668PyroF-BIO/rs4354668PyroR were used to generate biotinylated PCR products flanking SNPs rs1835740 and rs4354668, respectively. Primers rs1835740PyroSeq and rs4354668PyroSeq were used for pyrosequencing. The target sequence and the order of nucleotide dispensation for each pyrosequencing assay are listed. In the dispensation order the nucleotides used as negative controls for pyrosequencing are underlined. In optimal pyrosequencing conditions these nucleotides are not incorporated into the target DNA sequence and thus their addition do not generate peak on the pyrogram (see also Figure 2). The nucleotide change in the target sequence is indicated in bold.

### DNA sequencing

Genotypes from pyrosequencing were confirmed by Sanger sequencing (using ABI 3730xl 96 capillary DNA Analyzers) at Eurofins MWG Operon (Ebesberg, Germany). Ten samples were randomly selected for sequencing and PCR products were purified using Wizard® SV Gel and PCR Clean-Up System (Promega, Southampton, UK) following the manufacturer’s instructions.

### Statistical analysis

Basic statistical data (mean, standard deviation, standard error) were derived using MS Excel. Statistical analysis was carried out using a standard student’s *t*-test in Microsoft Excel™.

## Results

### Assessment of gDNA quantity and quality

Genomic DNA concentration and quality was determined in 177 samples using spectrophotometry (Table 1). The average concentration of gDNA was the highest in the umbilical cord extractions (UC) followed by the whole blood (WB) and then newborn DBS. A significant difference was observed between the three groups (Table 1; p < 0.01 WB *versus* UC; p < 0.001 newborn DBS *versus* UC; p < 0.001 WB *versus* newborn DBS). There was no significant correlation between the storage length and gDNA concentration in the DBS samples (Table 2).

The quality of gDNA was comparable between whole blood, umbilical cords and DBS prepared 1 month prior to extraction. The average A_260_:A_280_ ratio of the gDNA in these samples (1.7-1.9) fell within the optimal range for gDNA purity (1.8-2.0). However, the purity of gDNA in the DBS samples decreased with the storage length from A_260_:A_280_ 1.7 to 2.4 (p < 0.05) over the 22 year-period (Table 2).

### Analysis of DNA quality by agarose gel electrophoresis

To detect gDNA degradation in various samples, agarose gel electrophoresis was carried out (Figure 2A&B). Representative samples from each group were analysed for gDNA purity. All WB and UC samples showed uniform electrophoretic mobility and gDNA appeared as a single, high-molecular-weight band >10 kb (Figure 2B) with no low-molecular-weight fragmented bands present which would indicate sample degradation. In contrast, there was no clearly defined band at 10 kb visible in the DBS samples and the DNA produced a smear of low-molecular-weight fragmented bands on the gel indicating DNA degradation (Figure 2A). High quality gDNA is expected to be mostly >10 kb.

### PCR amplification of gDNA

PCR was performed to confirm the integrity of the gDNA and to determine if any inhibitory materials (e.g. guanidium, RNA or proteins) were present in the extractions. For this purpose a 325 bp fragment of a house keeping gene, β-actin was amplified which showed a clear specific band with the expected size (Figure 2C). All tested samples produced an amplicon at the expected size.

### Assessment of the fidelity of gDNA obtained from umbilical cords

**Figure 3:**
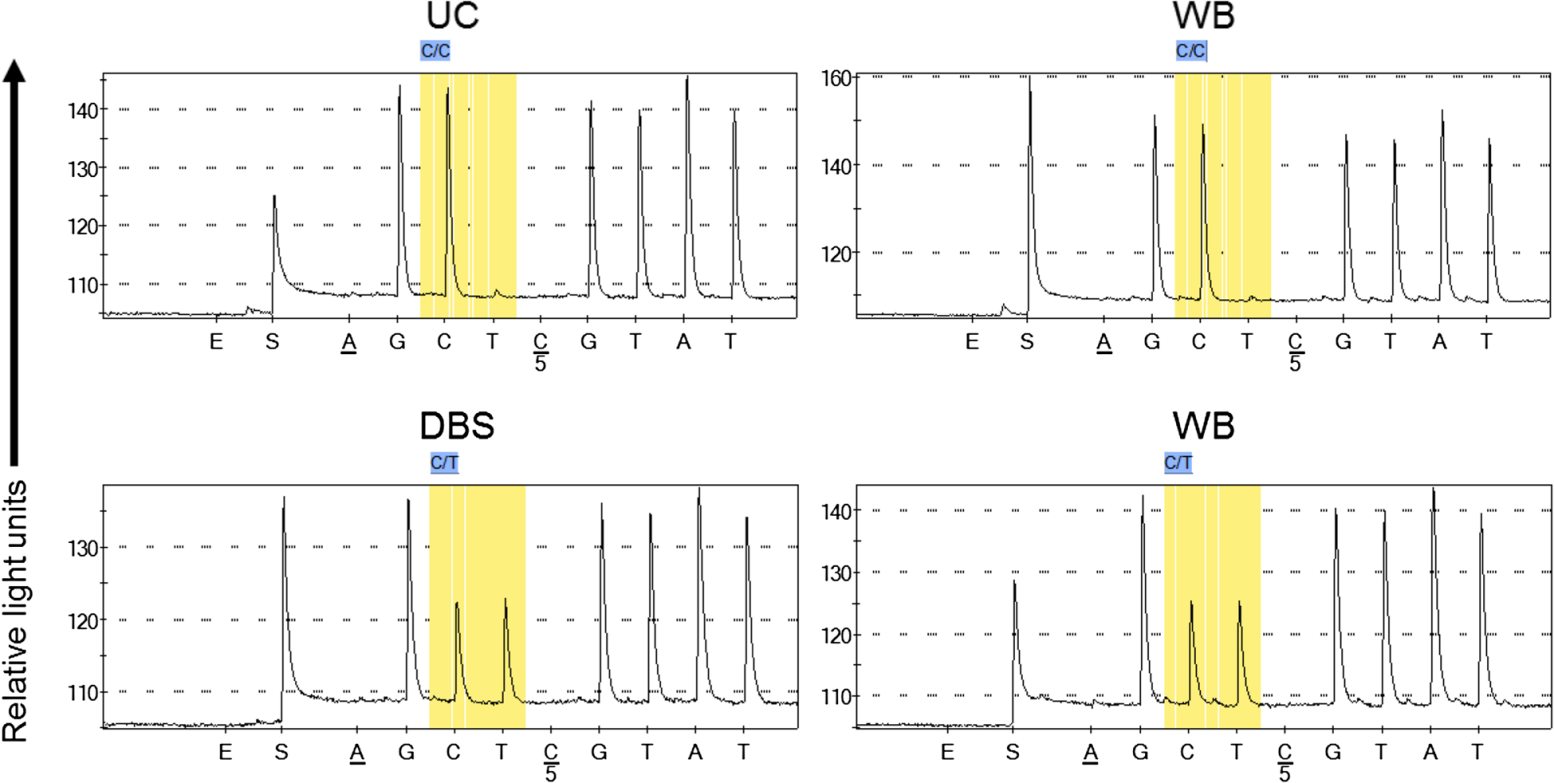
**Pyrograms showing genotyping of SNP rs1835470 using paired umbilical cord (UC) and whole blood (WB) gDNA (top panels) or dried blood spot (DBS) and whole blood (WB) gDNA (bottom panels) from the same individual.** The position of the SNP is highlighted in yellow boxes. Peak height is shown on the y-axes and the first nucleotide A and the fifth nucleotide C are negative controls and should not be incorporated into the target DNA sequence. E and S indicate enzyme and substrate, respectively.

As a further test for the quality of the gDNA extracted from umbilical cords, the genotype concordance between the umbilical cord gDNA and whole blood gDNA samples from the same individual were examined as a measure of the accuracy and hence reproducibility of the genotype calling. Thirty one individuals, where both umbilical cords and whole blood were available, were genotyped for two polymorphisms, SNP rs1835740 [26] and SNP rs4354668 [27] using pyrosequencing assays (Figure 3). The concordance rate for both SNPs between the two starting materials in each individual was a 100% (Table 4). The C allele frequency for SNP rs4354668 was high (0.42; Table 5) in the entire cohort (n = 656) and all genotypes were correctly detected in the UC samples indicating that no maternal gDNA contamination occurred. An example pyrogram is shown on Figure 3 for SNP rs1835740, where both umbilical cord and whole blood were used from the same individual for genotyping. The pyrogram illustrates that the signal strength and definition of peaks are very similar from both sources and the same genotype was obtained for both (Figure 3, top panels). Similarly, identical pyrograms were obtained for DBS and WB (Figure 3, bottom panels). The intensity of the signal generated from all three sources was comparable (Figure 3).

**Table 4:** The number of samples successfully genotyped/the total number of samples attempted for each SNP tested

**SNP**	**Sample call rate**	**Sample call rate**	**Sample call rate**
	**DBS**	**WB**	**UC**
**rs1835740**	682/723 (94%)	31/31 (100%)	31/31 (100%)
**rs4354668**	625/723 (86%)	31/31 (100%)	31/31 (100%)

**Table 5:** Distribution of alleles in the sample cohort

**SNP**	**WT/WT**	**WT/MT**	**MT/MT**	**Mutant allele frequency**
**rs1835740**	60%	33%	7%	0.23
**rs4354668**	30%	57%	13%	0.42

Distribution of the three genotypes (WT/WT, WT/MT, MT/MT in %) for SNPs rs1835740 and rs4354668 is shown. The frequency of the mutant allele (T allele for rs1835740 and C allele for rs4354668) is indicated in the last column.

## Discussion

Genetic analysis in premature infants is hampered by the very limited availability of samples suitable for gDNA extraction. While whole blood is rarely collected and stored for a long period from premature infants, newborn DBS are routinely obtained from all newborns 5-8 days after birth. These samples are often linked to databases which contain information on clinical outcomes for patients and gDNA can easily and quickly be extracted. However, in some biobanks (e.g. in the UK) newborn DBS are not readily available for academic research purposes within 3 years of collection because these samples may need to be recalled for further tests by the clinical pathology laboratory. Furthermore, DBS is not collected from babies who die within the first 4-5 days of life which could have significant impact on association studies investigating the impact of prematurity for example on brain injury. Similarly to newborn DBS, umbilical cord tissue could potentially be available for all newborns if appropriate ethical approvals are in place. The notable advantages of umbilical cord tissue are that *i)* it is available at birth for all infants; *ii)* its collection is independent of mortality rate which is significant in the very low birth weight preterm cohort [8]; *iii)* it can be collected non-invasively following birth and stored at -20°C until gDNA preparation and *iv)* it can provide good yield and high quality gDNA. This study aimed to assess the suitability of newborn DBS and umbilical cord tissue extracted gDNA as an alternative to venous blood-derived gDNA from premature neonates for genetic analysis.

The yields of gDNA extracted from whole blood and umbilical cords are comparable to previous studies where ∼6 μg gDNA/200 μl whole blood [28], ~100 μg gDNA/ 200 μl umbilical cord blood [29] were obtained (Table 2). The gDNA yield of DBS 180 ng/3.2 mm punch however was higher than previously published ~60 ng gDNA/3.2 mm punch [19] or 19-40 ng/3.0 mm punch [30]. These differences are most likely due to the non-uniform distribution of the blood on the card and the type of filter paper used for blood collection [30]. Indeed, blood spots which were not correctly collected had to be used for research purposes leaving the correctly collected blood spots for further clinical pathology investigations. It is unlikely that a pathological increase in white blood cells in the premature infants would be responsible for the increased gDNA yield observed in our study because a similar yield was achieved for both adults and newborns (Table 2, 1 month *versus* DBS samples 3-22 years). The gDNA extraction method can also have a significant impact on the yield. Carrier RNA was added to Buffer AL (Table 1), which enhances gDNA binding to the QIAamp column membrane, especially if there are very few target molecules in the sample. To further enhance gDNA binding, the column membrane was equilibrated with nuclease free water and the bound gDNA was eluted in two steps by adding 30 μl of nuclease free water twice.

The quality of gDNA from umbilical cord and newborn DBS was comparable to whole blood gDNA (1.7 and 1.9 *versus* 1.8, Table 1). A good quality gDNA sample should have an A_260_:A_280_ ratio between 1.7-2.0 [31,32]. In addition to measuring the A_260_:A_280_ ratio, a random selection of samples were analysed on agarose gels to eliminate the possibility of contaminants in the samples (i.e. guanidium, RNA or proteins; [31-34]; Figure 2A&B). These contaminants as well as degraded gDNA migrate at different rates compared to intact gDNA and thus can be detected on an agarose gel. No obvious contamination of gDNA was observed in the WB and UC samples (Figure 2B).

The length of storage of the dried blood spots did not significantly affect the total amount of gDNA recovered (Table 2). In contrast, the purity reduced significantly with storage length from 1.7 to 2.4 (Table 2). This is in line with previous studies that showed reduced gDNA quality following 25 years storage [35,36]. Similarly to our observation, even after 6 years of storage at room temperature the gDNA quality was reduced [35,36]. However, others reported that gDNA is stable for at least 11 years at ambient tropical conditions [37]. It is well documented that there are several factors that may compromise sample integrity which includes high humidity, temperature, persistence of nucleases and other chemical agents as well as other sub-optimal conditions that may occur not only during transport, but also within storage facilities [38]. Dry storage of nucleic acids has been recommended to eliminate the need for cold storage based on the assumption that nucleic acids are stable when dry. However there are numerous examples where degradation occurs during storage, in the cold or at ambient conditions, that can irreversibly damage samples in solution or even those that are dehydrated [39]. Although dried blood spots provide a valuable bioresource for research, DNA from this source has been shown to deteriorate with prolonged storage [40] which is in line with our observation. It has also been reported that the collection filter paper might have an impact on gDNA quality [30,35], but unfortunately there is no information available on the type of filter paper used for the collection of our samples or whether more than one type has been used.

To test the ability to detect the short DNA fragment of the β-actin gene in the samples, PCR amplification was used (Figure 2C). All whole blood, umbilical cord and DBS samples amplified β-actin successfully. All of these samples were then used to detect two unrelated SNPs by pyrosequencing (Figure 3). No direct link was observed between storage length and positive outcome with either PCR or pyrosequencing. While all of the samples from whole blood or umbilical cord produced conclusive pyrograms (Table 4), 6% and 14% of the DBS samples were unsuccessful for the detection of rs1835740 [26] and rs4354668 [27], respectively (Table 4). However, different samples failed the two PCR and pyrosequencing assays suggesting that the source of gDNA played an important role in the success of the analysis and the storage length did not seem to have a major impact. This is in line with previous observations [37,41] that gDNA fragmentation over time with storage has little impact on short DNA detection (200-700 bp). The variation observed in the PCR success rate might be dependent on the amount of natural PCR inhibitors (protein, haemoglobin, iron) present in the newborn DBS [40]. The concordance rate for both SNPs in gDNA prepared from umbilical cord tissue and whole blood was 100% (Table 4). The minor allele frequency for SNP rs4354668 was high in our premature infant cohort (0.42; Table 5) and all genotypes were correctly detected in the UC samples indicating that no maternal gDNA contamination occurred.

## Conclusions

This study established that both umbilical cord tissue and newborn DBS can be used as alternatives to whole blood for gDNA extraction from premature infants with suitable quality and fidelity for standard PCR and pyrosequencing-based genotyping. Considering the numerous advantages of using umbilical cord tissue for gDNA extraction, as discussed above, this could potentially improve recruitment to clinical studies and reduce ethical and logistical challenges associated with blood sample collection across multicentre studies. The quality and yield of gDNA from umbilical cord tissue makes it highly suitable for genome wide studies.

## Abbreviations

DBS: Dried blood spots; DNA: Deoxyribonucleic acid; gDNA: Genomic deoxyribonucleic acid; PCR: Polymerase chain reaction; kb: Kilobase; RNA: Ribonucleic acid; SNP: Single nucleotide polymorphism; K2-EDTA: Potassium ethylene diamine tetraacetic acid; Tris-HCI: Tris(hydroxymethyl)aminomethane hydrochloride; KCl: Potassium chloride; MgCl2: Magnesium chloride; dNTP: Deoxyribonucleotide triphosphate.

## Competing interests

The authors declare that they have no competing interests.

## Authors’ contributions

SR designed and carried out all experimental work. KL organised research ethics and NHS R&D permissions, parent information and consenting processes and clinical data collection. KL and ME collected the whole blood and umbilical cord tissue; KL and HK provided the DBS; MW assisted with the pyrosequencing analysis. EM and AV advised on experimental design. SR and AV wrote the manuscript and all authors reviewed the manuscript prior to submission. All authors read and approved the final manuscript.
